# New data from Monoplacophora and a carefully-curated dataset resolve molluscan relationships

**DOI:** 10.1038/s41598-019-56728-w

**Published:** 2020-01-09

**Authors:** Kevin M. Kocot, Albert J. Poustka, Isabella Stöger, Kenneth M. Halanych, Michael Schrödl

**Affiliations:** 1 Department of Biological Sciences and Alabama Museum of Natural History, University of Alabama, Tuscaloosa, Alabama 35487 USA; 2 Evolution and Development Group, Max-Planck Institute for Molecular Genetics, Berlin, 14195 Germany; 3 Environmental and Phylogenomics Group, Dahlem Centre for Genome Research and Medical Systems Biology, Berlin, 12489 Germany; 4 SNSB-Bavarian State Collection of Zoology, Munich, 81247 Germany; 5 Department Biology II, Ludwig-Maximilians-Universität, Planegg-Martinsried, 82152 Germany; 6 Department of Biological Sciences, Auburn University, Auburn, Alabama 36849 USA; 7 GeoBio-Center LMU, München, 80333 Germany

**Keywords:** Zoology, Phylogenetics

## Abstract

Relationships among the major lineages of Mollusca have long been debated. Morphological studies have considered the rarely collected Monoplacophora (Tryblidia) to have several plesiomorphic molluscan traits. The phylogenetic position of this group is contentious as morphologists have generally placed this clade as the sister taxon of the rest of Conchifera whereas earlier molecular studies supported a clade of Monoplacophora + Polyplacophora (Serialia) and phylogenomic studies have generally recovered a clade of Monoplacophora + Cephalopoda. Phylogenomic studies have also strongly supported a clade including Gastropoda, Bivalvia, and Scaphopoda, but relationships among these taxa have been inconsistent. In order to resolve conchiferan relationships and improve understanding of early molluscan evolution, we carefully curated a high-quality data matrix and conducted phylogenomic analyses with broad taxon sampling including newly sequenced genomic data from the monoplacophoran *Laevipilina antarctica*. Whereas a partitioned maximum likelihood (ML) analysis using site-homogeneous models recovered Monoplacophora sister to Cephalopoda with moderate support, both ML and Bayesian inference (BI) analyses using mixture models recovered Monoplacophora sister to all other conchiferans with strong support. A supertree approach also recovered Monoplacophora as the sister taxon of a clade composed of the rest of Conchifera. Gastropoda was recovered as the sister taxon of Scaphopoda in most analyses, which was strongly supported when mixture models were used. A molecular clock based on our BI topology dates diversification of Mollusca to ~546 MYA (+/− 6 MYA) and Conchifera to ~540 MYA (+/− 9 MYA), generally consistent with previous work employing nuclear housekeeping genes. These results provide important resolution of conchiferan mollusc phylogeny and offer new insights into ancestral character states of major mollusc clades.

## Introduction

Mollusca is the second most diverse animal phylum whose members exhibit an incredible array of body shapes and sizes. Many molluscs have important ecological roles in marine, freshwater, and terrestrial environments and others are culturally and/or economically important as a source of food, jewellery, or dye^1^. Despite their diversity and importance, understanding of early molluscan evolution remains incomplete and several conflicting phylogenetic hypotheses^1–9^ have been proposed regarding relationships among the eight major clades (i.e., classes): Bivalvia (clams, scallops, oysters, etc.), Caudofoveata (Chaetodermomorpha), Cephalopoda (octopuses, squids, and *Nautilus*), Gastropoda (snails and slugs), Monoplacophora (Tryblidia; deep-sea, limpet-like molluscs), Polyplacophora (chitons), Scaphopoda (tusk shells), and Solenogastres (Neomeniomorpha).

Within Conchifera (Bivalvia, Cephalopoda, Gastropoda, Monoplacophora, and Scaphopoda), the clade of molluscs with uni- or bivalved shells, the deep-sea limpet-like Monoplacophora has long been thought to be important to understanding early molluscan evolution^5,10–14^ with most morphology-based hypotheses placing Monoplacophora sister to a clade of all other conchiferans. However, no published molecular studies have supported this topology to date (but see Philippe and Roure 2012^15^). Studies of molluscan phylogeny employing datasets dominated by nuclear ribosomal and mitochondrial genes have generally had poor resolution among major lineages^10–14^. However, one finding of particular interest from these studies was the recovery of a close relationship of Monoplacophora and Polyplacophora (Serialia)^16,17^. More recent studies employing PCR-amplified fragments of nuclear protein-coding “housekeeping” genes^18^ or nuclear protein-coding genes obtained from transcriptome and genome data^19,20^ have instead provided strong support for a clade called Aculifera, which groups Polyplacophora with Aplacophora (Caudofoveata + Solenogastres) to form a group of molluscs with calcareous sclerites.

Smith *et al*.^19^, the only published phylogenomic study to date focused on deep molluscan relationships to sample Monoplacophora (specifically *Laevipilina hyalina*), recovered it as the sister taxon of Cephalopoda. This result is inconsistent with the prevailing traditional morphological view placing Monoplacophora sister to all other conchiferans^3,21–23^, but is consistent with some (but not all) palaeontological hypotheses on early molluscan diversification^24–27^. Two subsequent studies included data from *L*. *hyalina* but focused on relationships within Gastropoda^28^ or Bivalvia^29^, and thus had limited taxon sampling outside of those clades. Kocot *et al*.^30^ focused on among-phylum relationships within Lophotrochozoa but had relatively broad sampling of Mollusca. Most of those analyses recovered Monoplacophora as the sister taxon of Conchifera or Cephalopoda, but support for its placement was generally weak. Phylogenomic studies have also supported a clade including Gastropoda, Bivalvia, and Scaphopoda, although there has been inconsistency in recovered relationships among these taxa^19,20,28,30^. Because conchiferan molluscs are well-represented in the early animal fossil record^31,32^, understanding their phylogeny has important implications for understanding early animal evolution and the identity of enigmatic fossil taxa hypothesized to be stem-group molluscs.

## Results and Discussion

We sequenced a draft genome for the monoplacophoran *Laevipilina antarctica*. Unfortunately, because of the small size of this species, there was only adequate material for paired-end Illumina sequencing library preparation with insufficient material for mate pair, long-read, or transcriptome library preparation using techniques available at the time that this work was conducted. This resulted in a rather fragmented genome assembly (427,488 contigs >500 bp; N50 = 2,167 bp; 1.26 Gbp total assembly size). Assessment of this assembly with BUSCO^33^ showed that it is rather incomplete with only 14.6% of the 978 metazoa_odb9 genes recovered as complete and another 17.9% recovered as fragmented. Nevertheless, aside from transcriptome data from *Laevipilina hyalina*, these represent the only available genome data from any monoplacophoran and are thus a valuable resource for testing the phylogenetic position of this group.

We curated a dataset of 257 genes totalling 54,596 amino acids in length with data from 49 taxa of which 32 represented ingroup species (Supplementary Table 1). Care was taken to exclude possible contamination and mistranslated sequence regions (see Methods) while minimizing the amount of missing data in the final matrix (27.86% missing data). Additionally, only genes with a sequence from *L*. *antarctica* were sampled. Phylogenetic analyses were conducted using maximum likelihood (ML) in RAxML 8^34^ with the best-fitting model for each gene, and in IQ-TREE using the posterior mean site frequency (LG + C60 + G + F) mixture model^35–37^. A Bayesian inference (BI) analysis was conducted in PhyloBayes MPI^38^ with the CAT-GTR mixture model^39^.

**Figure 1:**
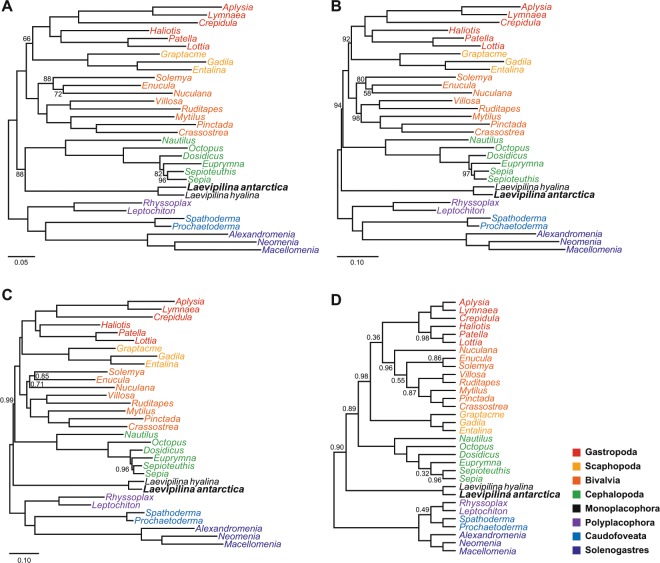
Results of phylogenetic analyses (outgroup taxa not shown). (**A**) RAxML maximum likelihood (ML) topology. Bootstrap support values below 100 shown. (**B**) IQ-TREE ML topology. Bootstrap support values below 100 shown. (**C**) PhyloBayes Bayesian inference (BI) topology. Posterior probabilities below 1.0 shown. (**D**) ASTRAL tree. Local posterior probabilities below 1.0 shown.

ML analysis of the partitioned dataset in RAxML (Fig. 1A) recovered Monoplacophora sister to Cephalopoda with moderate bootstrap support (bs = 88), consistent with the results of Smith *et al*.^19^ and some interpretations of the fossil record^11^. However, the ML analysis in IQ-TREE using the PMSF model (Fig. 1B) and the Bayesian inference analysis in PhyloBayes using the CAT-GTR model (Fig. 1C) recovered Monoplacophora sister to the rest of Conchifera with a bootstrap support value of 94 and posterior probability of 0.99 respectively, consistent with most morphology-based hypotheses of conchiferan relationships^11^.

To examine support for Monoplacophora sister to Conchifera from individual partitions, we used a multi-species coalescent approach in ASTRAL 5.6.1^40^. This analysis also recovered Monoplacophora sister to the rest of Conchifera (local posterior probability, lpp = 0.89; Fig. 1D).

Placement of Monoplacophora sister to all other conchiferans had a lower likelihood score than Monoplacophora + Cephalopoda in the RAxML analysis and could not be rejected by the Shimodaira-Hasegawa (SH) test (p = 0.190). This alternative topology was, however, rejected by the Approximately Unbiased (AU) test (p = 0.001). Both tests rejected the Serialia hypothesis (AU test p = 0.001; SH test p = 0).

A clade of all conchiferans except Monoplacophora, as recovered in most of our analyses, was originally proposed by morphologists and called Ganglionata (reviewed by Schrödl and Stöger 2014^5^). Despite the name, ganglia are neither restricted to Ganglionata nor do all species within Ganglionata show distinct pairs of ganglia^41–43^. Kocot *et al*.^20^ curated a morphological character matrix for Mollusca building on that of Haszprunar^21^ and conducted ancestral state reconstruction for key molluscan characters (see Methods) under a number of different phylogenetic scenarios including Monoplacophora sister to the rest of Conchifera. Our analyses placing Monoplacophora sister to the rest of Conchifera indicate that the only unambiguously apomorphic trait of Ganglionata is the reduction of adult dorsoventral muscle pairs from a hypothesized ancestral set of eight (or possibly seven^44^). Monoplacophorans also differ from other conchiferans with respect to the arrangement and structure of mantle folds, anatomy of the shell gland, and structure of the shell^23^, but whether these are retained conchiferan plesiomorphies or monoplacophoran apomorphies is ambiguous.

Relationships among Gastropoda, Bivalvia, and Scaphopoda, a clade of molluscs with relatively thick, multi-layered shells^27^, have been the subject of debate^3,5,7,8,31^ due to incongruence among recent studies^18–20,45,46^. Whereas our RAxML and ASTRAL analyses found poor support for relationships among these taxa, our IQ-TREE and PhyloBayes analyses using mixture models strongly supported Scaphopoda + Gastropoda with this clade sister to Bivalvia, consistent with Smith *et al*.^19^. Gastropoda is an extremely diverse, morphologically disparate, and ecologically variable group of species that inhabit almost all environments on land and in the sea. Scaphopoda, on the other hand, is a much less diverse group of relatively morphologically uniform animals that dig in marine sediments and prey upon foraminiferans and other infauna. This pair of unequal sister taxa contradicts the Cyrtosoma concept uniting Gastropoda and Cephalopoda (plus Monoplacophora by the original definition^10^; reviewed by Kocot^22^). Interestingly, a close relationship of Scaphopoda and Gastropoda was proposed based on the pronounced dorsoventral axis^47^ and recent work has confirmed the morphological ventral position of the scaphopod foot^48^. Examination of published molluscan morphological data matrices^20,21,49^ reveals obvious symplesiomorphies shared between these taxa (e.g., external univalved shell), but we find no clear morphological synapomorphies for the gastropod-scaphopod clade.

Consistent with other phylogenomic studies^18–20,50^, all of our analyses strongly support a molluscan dichotomy with two major clades: Conchifera and Aculifera^51^. Within Aculifera, we recovered chitons (Polyplacophora) sister to the vermiform, shell-less aplacophorans. Within Aplacophora, we recovered Solenogastres and Caudofoveata reciprocally monophyletic. Aculifera contradicts the classical morphology-based Testaria hypothesis^5^, which places chitons sister to Conchifera and the shell-less worm-like aplacophorans as an early-branching, paraphyletic grade. The Testaria hypothesis implies a progressive evolution from a simple unshelled worm-like ancestor towards chitons with shell plates and later with the uni- or bivalved conchiferans as the crown-group of Mollusca. Our results unequivocally reject this hypothesis (AU test p-value = 4.00E-56; SH test p-value = 0).

In light of support for placement of Monoplacophora sister to the rest of Conchifera and our earlier ancestral character state reconstruction analyses based on this phylogenetic hypothesis^20^, we infer that the last common ancestor of extant molluscs was likely a dorsoventrally flattened animal that had a mantle, a dorsal cuticle, a broad foot, eight (or seven^44^) pairs of dorsoventral muscles, a circumpedal or posterior mantle cavity with serially arranged gills, and a radula as part of a longitudinally arranged, regionalized digestive system. Whether or not the last common ancestor of extant molluscs had a single shell, multiple shell plates, or no shell is ambiguous^20^. Possession of a single shell is clearly plesiomorphic for Conchifera but this was probably also the case in *Calvapilosa*, *Maikhanella*, and *Orthrozanclus*, fossil taxa inferred to be stem aculiferans^52^, suggesting that the last common molluscan ancestor may have been single-shelled. Additional studies comparing development, mineralogy, and other structural aspects of chiton shells, conchiferan shells, and aculiferan sclerites would be of great interest to further address this and other important questions about the origin(s) and homology of molluscan biomineralized structures^53^.

**Figure 2:**
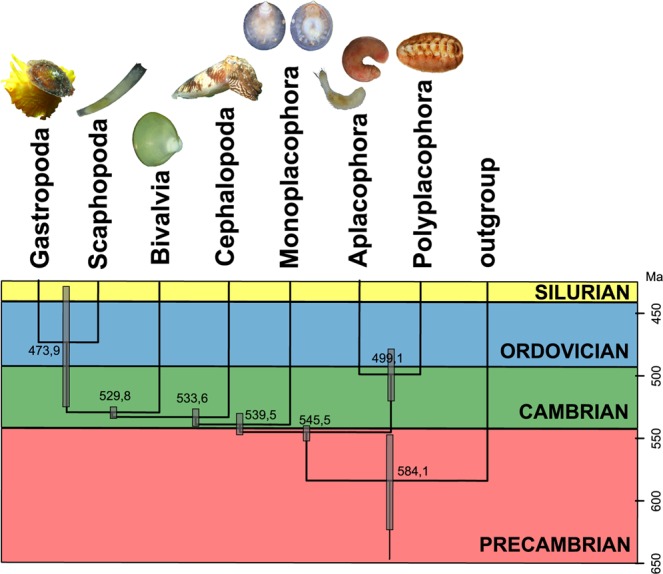
Summary of relaxed molecular clock analysis results. Numbers along y-axis are millions of years before present (Ma). Numbers at nodes represent the average age of the split; Error bars at nodes represent the height 95% HPD (highest posterior density). A detailed version of this tree is presented in Supplementary Fig. 1 and the raw data and uncollapsed tree are available via FigShare (see Data Availability section).

Our molecular clock analysis (Fig. 2; Supplementary Fig. 1; Supplementary Table 3) indicates that the molluscan stem split from trochozoan relatives about 584 MYA (95% highest posterior density [HPD] = 547–623 MYA), Conchifera diversified 540 MYA (531–548 MYA), and Aculifera diversified 499 MYA (479–520 MYA), generally consistent with previous relaxed molecular clocks calculated from multilocus^18,45,52,54^ and phylogenomic data^28,55^, showing the molluscan stem to be Precambrian in origin. The Ediacaran fossil genus *Kimberella* has been hypothesized to represent a stem-group mollusc by some^31,56–58^ but the molluscan affinity of *Kimberella* has been criticized by others who instead view it as an early-branching bilaterian^32^, in part because of its old age (~555 MYA). Although broad, our and other recent estimates for the divergence of molluscs are at least compatible with hypotheses regarding *Kimberella* as an early offshoot of the molluscan stemline^31,45,59^. However, if *Kimberella* was indeed a mollusc, it differed from most extant molluscs in its lack of a shell (although sclerites may have been present) and, more significantly, a bizarre rake-like mode of feeding unlike that of any modern mollusc^32^.

Late Precambrian and Cambrian small shelly fossil (SSF) assemblages consist of abundant, diverse, and tiny (0.5–5 mm) animals^60 in strong contrast to the large-bodied Vendian *Kimberella*^. Our time tree is consistent with the prevailing notion that SSFs such as helcionellids and other gastropod- and monoplacophoran-like fossils were conchiferan molluscs^32^, but relatively broad posterior densities preclude confident placement of these fossil taxa along any one branch. According to our time tree, molluscan SSFs would have been stem conchiferans, or less likely, belonged to the stem of Monoplacophora or the lineage that gave rise to the remaining conchiferans. As noted above, at least some fossil aculiferans had a single shell; at least some SSFs could conceivably have been aculiferans. Surprisingly, the split of gastropods and scaphopods is rather late according to our molecular clock analysis (474 MYA; 95% HDP = 479–520 MYA); this could mean that many Cambrian shells currently regarded to be gastropods were actually members of the gastropod-scaphopod stem lineage.

In conclusion, we analysed a high-quality and representative molluscan phylogenomic dataset and recovered a robust and intriguing hypothesis on molluscan class-level relationships. Analyses employing site-heterogeneous models and a coalescent approach provide support for a dichotomy dividing the molluscs into Aculifera and Conchifera, the latter with Monoplacophora sister to the rest of uni- or bivalved molluscs and gastropods sister to scaphopods, not bivalves. Our results contradict hypotheses such as Testaria, Serialia, and Monoplacophora + Cephalopoda, and have important consequences for reconstructing early molluscan evolution.

## Methods

### Molecular laboratory work

One specimen of *Laevipilina antarctica* (ZSM-Mol-20090330, DNABANK-Mol-MS-016) was collected with the *R/V Polarstern* in Antarctica between 70°24.00′S, 8°19.72′W and 70°23.86′S, 8°18.68′W at 597–602 m depth on 12 January 2008. DNA was extracted from the specimen using the NucleoSpin Tissue Kit (Macherey-Nagel, Düren, Germany). DNA (10 ng) was used for whole genome amplification using the Illustra GenomiPhi V2 DNA Amplification Kit (GE Healthcare Life Sciences, Freiburg, Germany) followed by standard ethanol precipitation and re-purification using the Qiagen MinElute system (Qiagen, Hilden, Germany). Concentration was determined using a Qubit 2.0 Fluorometer, and 1 μg was used to create a sequencing library with the TruSeq DNA Sample Preparation Kit v2 (Illumina, San Diego, CA, USA) with an average insert size of approximately 250 bp. Two lanes of 101 bp paired-end-reads were sequenced on the Illumina HiSeq 2000 system yielding about 90 Gbp. Raw reads were filtered for quality, PCR duplicates, and adapter sequences and corrected using SOAPfilter_v2.0 (https://github.com/tanghaibao/jcvi-bin/blob/master/SOAP/SOAPfilter_v2.0) using default settings.

### Genome assembly and annotation

Reads retained by SOAPfilter_v2.0 were assembled *de novo* using SOAPdenovo2_v2.04^61^. Sparse_pregraph was used to construct the K-mer graph using the following settings: -K 31 -g 15 -z 2000000000 -d 1 -e 1 -r 0 -p 28. Contigs were computed using kmer iterations up to K = 63 (-M 3 -m 63 -p 30). The remapping step of SOAPdenovo was carried out using standard settings and the scaffolding step was used with parameters: -F -G 200 -p 28. Finally, additional gaps were filled using SOAP Gapcloser v1.12. Genescan^62^ was used to generate gene predictions resulting in 83 Mb of protein-coding sequences, which were subsequently used for phylogenomic analyses.

### Taxon sampling and data preparation for phylogenomic analysis

Taxon sampling (Supplementary Table 1) was selected to broadly span the diversity of Mollusca including at least two representatives of each major lineage and at least two representatives of each phylum considered a candidate for the sister taxon of Mollusca^63^. Publicly available protein sequences from complete genomes and assembled transcriptomes were downloaded when available. Dataset assembly and processing built on our established and routinely used bioinformatic pipeline^30,64–67^ with a number of modifications to help reduce possible exogenous contamination and low quality data (e.g., incorrectly translated gene predictions from Genescan; see below). Unassembled publicly available transcriptome data were digitally normalized and assembled using Trinity^68^. Transcriptome assemblies were translated with TransDecoder (https://sourceforge.net/p/transdecoder/), keeping only amino acid (AA) sequences longer than 100 AAs.

### Orthology inference

For orthology inference, we employed HaMStR 13^69^, which infers orthology based on predefined sets of orthologous groups (OGs). We employed the Trochozoa custom core-ortholog set of Kocot *et al*.^30^. Translated transcripts for all taxa were then searched against the 2,259 Trochozoa pHMMs. Sequences matching an OG’s pHMM were then compared to the proteome of *Lottia gigantea* using BLASTP^70^ with the -strict option. If the *Lottia* amino acid sequence contributing to the pHMM was the best BLASTP hit in each of these back-BLASTs, the sequence was then assigned to that OG.

### Dataset processing

Sequences shorter than 100 amino acids were deleted and OGs sampled for fewer than 35 taxa were discarded. Redundant identical sequences were removed with UniqHaplo (http://raven.iab.alaska.edu/~ntakebay/). In cases where one of the first or last 20 characters of an amino acid sequence was an X, all characters between the X and that end of the sequence were deleted and treated as missing data. Each OG was then aligned with MAFFT^71^ (mafft–auto–localpair–maxiterate 1000). Alignments were then trimmed with Aliscore^72^ and Alicut^73^ to remove ambiguously aligned regions. Next, a consensus sequence was inferred for each alignment using the EMBOSS program infoalign^74^. For each sequence in each single-gene amino acid alignment, the percentage of positions of that sequence that differed from the consensus of the alignment were calculated using the infoalign’s “change” calculation. Any sequence with a “change” value greater than 75 was deleted. Subsequently, a custom script (AlignmentCompare; https://github.com/kmkocot/basal_metazoan_phylogenomics_scripts_01-2015) was used to delete any likely mistranslated sequence regions of 20 or fewer amino acids in length surrounded by ten or more gaps on either side. Next, alignment columns with fewer than four non-gap characters were deleted. At this point, alignments shorter than 50 amino acids in length were discarded. Lastly, sequences that did not overlap with all other sequences in the alignment by at least 20 amino acids were deleted, starting with the shortest sequences not meeting this criterion.

In some cases, a taxon was represented in an OG by two or more sequences (splice variants, lineage-specific gene duplications [=inparalogs], overlooked paralogs, or exogenous contamination). In order to select the best sequence for each taxon and exclude any paralogs or exogenous contamination, we built trees in FastTree 2^75^ and used PhyloTreePruner^76^ to select the best sequence for each taxon. OGs sampled for fewer than 35 taxa and OGs lacking a sequence from *Laevipilina antarctica* were discarded. The remaining alignments were manually screened to identify and remove putative contamination or mistranslated sequences. Sequences that were obviously very different from the majority of the sequences in the alignment were blasted against NCBI NR using BLASTP and sequences that did not return an animal as the top hit were discarded. Finally, remaining OGs were then concatenated using FASconCAT^77^.

### Phylogenetic analyses

Maximum likelihood analyses were conducted in RAxML 8.2.4^34^ and IQ-TREE 1.5.5^35^. For the RAxML analysis, matrices were partitioned by gene with the PROTGAMMAAUTO model (the best-fitting model for each gene) used for all partitions. The tree with the best likelihood score after 10 random addition sequence replicates was retained and topological robustness (i.e., nodal support) was assessed with 100 replicates of fast bootstrapping (the -f a command line option was used). For the IQ-TREE analysis, we used the posterior mean site frequency (PMSF) model^37^, which is an approximation to full empirical profile mixture models for ML analysis. Specifically, the LG + C60 + G + F model was specified. Because this approach requires a guide tree to infer the site frequency model, we used the previously generated RAxML tree. Nodal support was assessed with 1000 replicates of ultrafast bootstrapping (-bb 1000). Bayesian Inference analysis was conducted with PhyloBayes 4.1b^78^ using the site-heterogeneous CAT-GTR model. Two chains were run for 14,143 and 13,400 generations, respectively with the first 2,000 trees from each chain discarded as burn-in. A bpcomp maxdiff value of 0.28 indicated that the chains had converged.

To examine support for key hypotheses from individual partitions, we made trees for each gene in RAxML using the best-fitting model, used these as guide trees for IQ-TREE analyses with the LG + C20 + G + F model, and inferred a supertree using a multi-species coalescent model in ASTRAL 5.6.1^40^. Weakly-supported nodes (bs < 50) were collapsed as advocated by Zhang *et al*.^40^. Hypothesis testing using the Approximately Unbiased test^79^ and the Shimodaira Hasegawa test^80^ was conducted using RAxML 8.2.4^34^ and CONSEL^81^ based on the RAxML analysis.

Divergence time estimates (Supplementary Table 3) were obtained in BEAST2 v.2.4.6^82^ on the CIPRES Science Gateway (https://www.phylo.org/) with a log-normal relaxed clock and the WAG model of substitution. The topology of the tree was manually constrained *a priori* by defining the major splits of the BI tree analysed herein. Fossil calibrations^83^–^89^ are presented in Supplementary Table 4. The analysis was executed for 180 million generations sampling a tree every 1,000 generations. After discarding the first 3,600 trees as burn-in, 14,401 trees were analysed with TreeAnnotator 2.4.5 to build the summary tree.

### Ancestral character state reconstruction

Ancestral character state reconstruction was performed previously by Kocot *et al*.^20^ using an updated and modified version of the morphological matrix of Haszprunar^21^. Because this analysis was already performed in light of numerous alternative hypotheses of molluscan class-level phylogeny including Monoplacophora sister to the remainder of Conchifera, it was not re-done here. The data matrix analysed is available via FigShare at https://figshare.com/s/934e61a053aacd8d37c1.

## Supplementary information

Supporting Information.

## Data Availability

Illumina paired-end genomic data for *L*. *antarctica* were submitted to NCBI SRA under accession number SRR6506080. The assembled *L*. *antarctica* genome, assembly statistics, Genescan output, molecular and morphological data matrices analysed, and other data files associated with results presented herein were submitted to FigShare: https://figshare.com/s/934e61a053aacd8d37c1. Sources of publicly available datasets used herein are presented in Supplementary Table 1.
