# Supplementary material for: New data from Monoplacophora and a carefully-curated dataset resolve molluscan relationships
Source: Sci Rep. 2020 Jan 9;10:101. doi: 10.1038/s41598-019-56728-w (PMC6952402; doi:10.1038/s41598-019-56728-w)
Supplement: Supplementary file 1 — Supporting Information. [file 41598_2019_56728_MOESM1_ESM.docx]

**Supplementary Information**

Supplemental Information includes one figure and four tables and can be found with this article online.


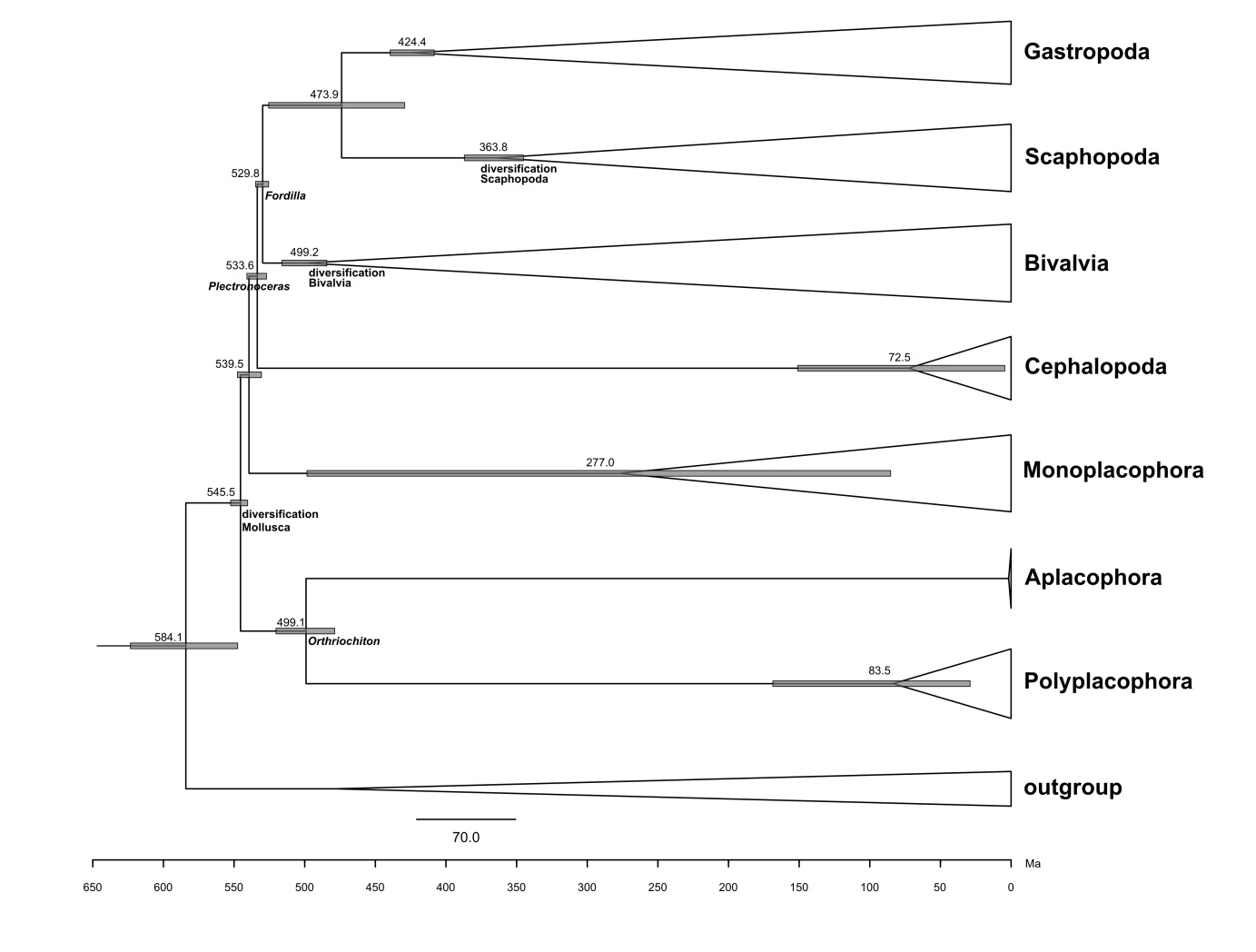


**Supplementary Figure 1.** Detailed presentation of results of relaxed molecular clock analysis (Figure 2). Numbers along x-axis are millions of years before present (Ma). Numbers at nodes represent the average age of the split; Error bars at nodes represent the 95% HPD (highest posterior density). The raw data and uncollapsed tree are available via FigShare (see Data Availability section).

**Tables**

**Supplementary Table 1.** Taxon sampling.

| **Taxon** | **Species** | **Abbrev.** | **Type** | **Reads** | **HaMStR genes** | **Source** | **Accession Number(s) / Version  / URL / Citation** |
| --- | --- | --- | --- | --- | --- | --- | --- |
| **Solenogastres** | *Alexandromenia crassa* | ACRA | Illumina | 45,059,456 | 2,084 | NCBI SRA | SRR2052564 |
|  | *Macellomenia schanderi* | MSCH | Illumina | 49,904,154 | 2,130 | NCBI SRA | SRR2057023 |
|  | *Neomenia carinata* | NCAR | Illumina | 36,612,396 | 1,547 | NCBI SRA | SRR2057026 |
| **Caudofoveata** | *Prochaetoderma californicum* | PCAL | Illumina | 90,561,352 | 2,116 | NCBI SRA | SRR6926326 |
|  | *Spathoderma clenchi* | SCLE | Illumina | 52,062,402 | 1,172 | NCBI SRA | SRR8258011 |
| **Polyplacophora** | *Leptochiton rugatus* | LRUG | Illumina | 49,670,054 | 2,096 | NCBI SRA | SRR1611558 |
|  | *Rhyssoplax olivaceus* | ROLI | Illumina | 23,189,291 | 2,149 | NCBI SRA | SRR618506 |
| **Gastropoda** | *Aplysia californica* | ACAL | Sanger | 216,556 | 931 | NCBI UniGene | January 27, 2010 Version |
|  | *Crepidula fornicata* | CFOR | Illumina | 39,362,017 | 1,883 | NCBI SRA | SRR1324873-SRR1324880 |
|  | *Haliotis rufescens* | HRUF | Illumina | 355,678,562 | 2,177 | Dryad | http://datadryad.org/resource/ doi:10.5061/dryad.85p80 |
|  | *Lottia gigantea* | LGIG | Genome | - | 2,259 | JGI | JGI filtered models v. 1.0 |
|  | *Lymnaea stagnalis* | LSTA | Illumina | 81,851,004 | 936 | NCBI SRA | DRR002012 |
|  | *Patella vulgata* | PVUL | Illumina | 47,237,104 | 1,931 | From authors | https://doi.org/10.1007/s10126-012-9481-0 |
| **Bivalvia** | *Crassostrea gigas* | CGIG | Genome | - | 2,199 | V9 protein models | http://gigadb.org/Pacific_oyster/ |
|  | *Enucula tenius* | ETEN | Illumina | 77,448,350 | 1,760 | Dryad – exemplars/isotigs only | dryad.34644 |
|  | *Mytilus edulis* | MEDU | Illumina | 72,220,824 | 2,238 | NCBI SRA | SRX565221-SRX565224 |
|  | *Nuculana pernula* | NPER | Illumina | 35,983,152 | 591 | NCBI SRA | SRR2057025 |
|  | *Pinctada fucata* | PFUC | Genome | - | 2,073 | From authors | http://marinegenomics.oist.jp/ genomes/download?project_id=20 |
|  | *Ruditapes philippinarum* | RPHI | Illumina | 41,031,443 | 1,911 | NCBI Nucleotide Database | JO101212-JO124029 |
|  | *Solemya velum* | SVEL | Illumina | 66,597,054 | 2,068 | Dryad – exemplars/isotigs only | dryad.34644 |
|  | *Villosa lienosa* | VLIE | Illumina | 162,000,000 | 2,118 | NCBI BioProject | PRJNA75063, ID #75063 |
| **Scaphopoda** | *Entalina tetragona* | ETET | Illumina | 39,609,424 | 1,575 | NCBI SRA | SRR2057018 |
|  | *Gadila tolmiei* | GTOL | Illumina | 75,942,132 | 1,815 | Dryad – exemplars/isotigs only | dryad.34644 |
|  | *Graptacme eborea* | GEBO | Illumina | 61,523,742 | 2,156 | NCBI SRA | SRR2057020 |
| **Monoplacophora** | *Laevipilina antarctica* | LANT | Genome | - | 2,233 | NCBI SRA | SRR6506080 |
|  | *Laevipilina hyalina* | LHYA | 454, Sanger | 75,485 | 430 | Dryad – transcripts | dryad.34644 |
| **Cephalopoda** | *Dosidicus gigas* | DGIG | Illumina | 37,094,323 | 1,663 | NCBI SRA | SRR1386212 |
|  | *Euprymna scolopes* | ESCO | Sanger | 35,420 | 1,179 | NCBI Trace Archive | DW251302-DW286722 |
|  | *Nautilus pompilius* | NPOM | 454 | 549,720 | 406 | NCBI SRA | SRR108979 |
|  |  |  | 454 | 112,375 | 464 | Dryad – exemplars/isotigs only | dryad.34644 |
|  | *Octopus vulgaris* | OVUL | Illumina | 16,501,336 | 1,742 | Dryad – exemplars/isotigs only | dryad.34644 |
|  | *Sepia esculenta* | SESC | Illumina | 80,947,907 | 1,705 | NCBI SRA | SRR1386223 |
|  | *Sepioteuthis lessoniana* | SLES | Illumina | 67,170,966 | 1,482 | NCBI SRA | SRR1386192 |
| **Annelida** | *Boccardia proboscidea* | BPRO | Illumina | 63,634,426 | 1,722 | NCBI SRA | SRR2057014 |
|  | *Capitella teleta* | CTEL | Genome | - | 2,215 | JGI | JGI v1.0 |
|  | *Clymenella torquata* | CTOR | Illumina | 85,285,816 | 2,129 | NCBI SRA | SRR2057016 |
|  | *Glycera dibranchiata* | GDIB | Illumina | 82,775,880 | 1,299 | NCBI SRA | SRR2057019 |
|  | *Helobdella robusta* | HROB | Genome | - | 2,049 | JGI | JGI filtered models v. 3 |
|  | *Pectinaria gouldii* | PGOU | Illumina | 145,853,782 | 1,506 | NCBI SRA | SRR2057036 |
|  | *Phascolosoma agassizii* | PAGA | Illumina | 63,918,870 | 1,876 | Dryad – transcripts | dryad.30k4v |
| **Brachiopoda** | *Hemithiris psittacea* | HPSI | Illumina | 60,731,022 | 2,224 | NCBI SRA | SRR1611556 |
|  | *Glottidia pyramidata* | GPYR | Illumina | 67,613,510 | 2,101 | NCBI SRA | SRR1611555 |
|  | *Laqueus californicus* | LCAL | Illumina | 67,414,776 | 2,113 | NCBI SRA | SRR1611557 |
|  | *Novocrania anomala* | NANO | Illumina | 52,243,928 | 1,531 | NCBI SRA | SRR1611564 |
| **Phoronida** | *Phoronis psammophila* | PPSA | Illumina | 58,372,182 | 2,223 | NCBI SRA | SRR1611565 |
|  | *Phoronis vancouverensis* | PVAN | Illumina | 69,531,036 | 2,211 | NCBI SRA | SRR1611566 |
| **Entoprocta** | *Barentsia gracilis* | BGRA | Illumina | 67,947,336 | 1,283 | NCBI SRA | SRR1611554 |
|  | *Loxosoma pectinaricola* | LPEC | Illumina | 75,025,552 | 1,675 | NCBI SRA | SRR1611559 |
| **Nemertea** | *Malacobdella grossa* | MGRO | Illumina | 30,538,858 | 1,878 | NCBI SRA | SRR1611560 |
|  | *Paranemertes peregrina* | PPER | Illumina | 59,441,992 | 1,986 | NCBI SRA | SRR1611562 |

**Supplementary Table 2.** Hypothesis test results.

| **Constraint** | **Log-likelihood** | **AU test  (p-value)** | **SH test  (p-value)** |
| --- | --- | --- | --- |
| Unconstrained | -1,481,197.46 |  |  |
| Monoplacophora sister to all other conchiferans | -1,481,202.46 | 0.001 | 0.190 |
| Gastropoda + Bivalvia | -1,481,209.21 | 3.00E-78 | 3.00E-04 |
| Diasoma | -1,481,227.52 | 0.198 | 0.814 |
| Patellogastropoda sister to clade of all other gastropods | -1,481,298.44 | 0.210 | 0.744 |
| Cyrtosoma (as Gastropoda + Cephalopoda) | -1,481,535.61 | 0.448 | 0.827 |
| Serialia | -1,481,747.97 | 0.001 | 0.000 |
| Testaria | -1,482,088.01 | 4.00E-56 | 0.000 |

**Supplementary Table 3.** BEAST2 molecular clock results. HPD = highest posterior density.

| **Node** | **Divergence time (Ma)** | **95% HPD (Ma)** |
| --- | --- | --- |
| Mollusca | 545.449 | 540.386 - 552.405 |
| Aculifera | 499.060 | 478.787 - 520.313 |
| Aplacophora | 1.677 | 1.527 - 1.912 |
| Solenogastres | 0.777 | 0.703 - 0.887 |
| Caudofoveata (Prochaetodermatidae) | 0.127 | 0.070 - 0.183 |
| Polyplacophora | 83.455 | 29.001 - 168.611 |
| Conchifera | 539.449 | 530.604 - 547.625 |
| Monoplacophora (*Laevipilina*) | 276.956 | 85.269 - 498.414 |
| Ganglionata | 533.595 | 527.025 - 540.918 |
| Cephalopoda | 72.505 | 4.478 - 150.959 |
| Bivalvia + Gastropoda + Scaphopoda | 529.765 | 525.529 - 534.738 |
| Bivalvia | 499.182 | 484.407 - 516.073 |
| Gastropoda + Scaphopoda | 473.888 | 429.253 - 525.494 |
| Scaphopoda | 363.764 | 345.457 - 386.955 |
| Gastropoda | 424.426 | 408.354 - 439.456 |
| Mollusc stem/Trochozoa | 584.137 | 547.438 - 623.354 |

**Supplementary Table 4.** Constraints used in molecular clock analysis.

| **Calibration node** | **Fossil calibration** | **Date range (Ma)** | **Reference** | **Prior settings in BEAST v.2.4.6 (distribution; gamma shape, gamma scale, zero offset)** |
| --- | --- | --- | --- | --- |
| Diversification of Mollusca | first shell record | ~ 545 | ^63^ | Gamma; 2.5, 2.0, 540.0 |
| Split of Bivalvia/Scaphopoda/Gastropoda | *Fordilla* | ~ 530 | ^63^ | Gamma; 3.3, 2.2, 525.0 |
| Split of Cephalopoda/Bivalvia/Scaphopoda/ Gastropoda | *Plectronoceras* | ~ 505 | ^86^ | Gamma; 2.4, 7.0, 495.0 |
| Split of Polyplacophora/ Aplacophora | *Orthriochiton* | ~ 490 | ^87^ | Gamma; 5.0, 5.0, 470.0 |
| Diversification of Bivalvia |  | ~ 490 | ^48^ | Gamma; 5.0, 5.0, 470.0 |
| Origin of Caenogastropoda | Sublitoidea | ~ 418 | ^88^ | Gamma; 2.3, 9.0, 405.0 |
| Diversification of Scaphopoda | *Dentalium* | ~ 353 | ^89^ | Gamma; 2.2, 6.7, 345.0 |
